# Patients With Cirrhosis Have Higher Costs of Care, Longer Length of Stays, and More Perioperative Complications Following Total Knee Arthroplasty: A National Inpatient Sample-Based Study

**DOI:** 10.7759/cureus.47317

**Published:** 2023-10-19

**Authors:** Sreenivasulu Metikala, Nikit Venishetty, Naga Cheppalli, Hunter Jones, Varatharaj Mounsamy, Senthil Sambandam

**Affiliations:** 1 Orthopaedics, Virginia Commonwealth University Health System, Richmond, USA; 2 Orthopaedic Surgery, Paul L. Foster School of Medicine, El Paso, USA; 3 Orthopaedics, Veterans Affairs (VA) Hospital Albuquerque, Albuquerque, USA; 4 Orthopaedic Surgery, University of Texas Southwestern Medical Center, Dallas, USA; 5 Orthopaedics, University of Texas Southwestern Medical Center, Dallas, USA

**Keywords:** cost of care, postoperative complications, length of stay, cirrhosis, total knee arthroplasty

## Abstract

Background

Cirrhosis is a growing disease affecting millions of people in the United States annually. Many cirrhosis patients undergo significant procedures and are met with increased risks such as encephalopathy, impaired immune response, ascites, variceal bleeding, renal disease, and increased malnutrition. Many cirrhosis patients need to undergo major surgical procedures such as total knee arthroplasty (TKA); however, perioperative complications following TKA in cirrhosis patients have not been studied. The purpose of this study was to analyze the demographic characteristics and perioperative complications of cirrhosis patients following TKA.

Methods

Using the National Inpatient Sample (NIS) database, we looked at retrospective data from the years 2016-2019 to analyze the incidence of perioperative complications, length of stay (LOS), and the cost of care (COC) among patients undergoing TKA who were categorized as cirrhosis patients, compared with those who are not. A propensity match was conducted to consider associated comorbidities that influence perioperative complications.

Results

Of the 558,256 patients analyzed who underwent TKA, 1670 (0.3%) were diagnosed with cirrhosis. After matching, cirrhosis patients had a longer LOS (4.22 vs. 3.68 days, p=0.016) and COC ($90,624 vs. 80676.87, p<0.001) than patients in the control group. Moreover, cirrhosis patients had a higher likelihood of developing acute renal failure (odds ratio (OR): 3.05, 95% CI: 2.07-4.50, p<0.001), blood loss anemia (OR: 1.60, 95% CI: 1.34-1.92, p<0.001), periprosthetic fracture (OR: 3.27, 95% CI: 1.31-8.18, p=0.007), periprosthetic infection (OR: 3.14, 95% CI: 1.99-4.95, p<0.001), and blood transfusions (OR: 1.62, 95% CI: 1.12-2.35, p=0.009) than patients in the control group.

Conclusion

The cirrhosis group had a significantly higher COC, longer LOS, and higher rates of perioperative complications than non-cirrhosis patients. This data will help providers make informed decisions about patient care and resource allocation for cirrhosis patients undergoing TKA.

## Introduction

Cirrhosis poses a significant surgical risk factor due to preexistent encephalopathy, coagulopathy, impaired immune response, malnutrition, osteopenia, ascites, variceal bleeding, renal disease, and infection [1,2]. The common etiological factors of cirrhosis include viral hepatitis, alcohol abuse, autoimmune hepatitis, biliary duct disease, genetic or metabolic disorders, non-alcoholic fatty liver disease (NAFLD), and prolonged exposure to toxins and industrial pollutants [3,4]. During the past decade, with the global burden of the disease gradually escalating, there have been substantial changes in its epidemiology [5-7]. Thus, North America is witnessing a surge in NAFLD and alcohol-related cirrhosis, while viral hepatitis remains the primary cause of cirrhosis worldwide [8-10]. As a chronic irreversible liver disease with multiorgan dysfunction, cirrhosis is associated with not just morbidity but also mortality. A total of 2.4% of global deaths in 2019 were attributed to cirrhosis, predicted to increase in the coming decade [9,10]. The Child-Pugh classification [11] is the go-to scoring system for assessing the overall prognosis and stratifying surgical risks. Three main classes, A, B, and C, are distinguished by increasing disease severity, and elective surgery like total knee arthroplasty (TKA) is usually well tolerated in class-A cirrhotic patients [12]. Notably, the rapid advances in medical management have led to a rise in the prevalence of cirrhosis, besides increasing the survival rates. Consequently, the utilization of TKA has risen substantially in this high-risk patient cohort with improving medical optimization [13,14]. It is widely recognized that cirrhotic patients tend to have poorer outcomes than the non-cirrhotic groups following major orthopedic surgeries [15-17]. Similarly, TKA in this sick patient population carries a distinct set of perioperative and postoperative complications [2,18,19]. Although data exist on the general risk factors, challenges, and outcomes of TKA in cirrhotic patients, little is known about immediate perioperative outcomes and their impact on hospital resources. This research was conducted to evaluate the association between cirrhosis and inpatient complications following TKA, together with the duration of hospital stay and healthcare costs, based on the recent four-year Nationwide Inpatient Sample (NIS) data.

## Materials and methods

Database description

The NIS contains information on more than 7,000,000 hospital stays and is the biggest all-payer, publicly accessible inpatient care database in the US [20]. The NIS database was used to get information on patients who received primary TKA between 2016 and 2019 in the US. Due to the enormous sample size, it offers the perfect information for creating national and regional estimations. It also makes it possible to analyze unusual pathologies, uncommon treatments, and particular demographics. The diseases are categorized using the International Classification of Disease-Tenth Revision, Clinical Modification/Procedure Coding System (ICD-10-CM/PCS) in the NIS database that was available between 2016 and 2019. The NIS database contains data on patient demographics, length of stay (LOS), payment source, hospital charges, discharge status, comorbidities, and several other variables. Data was extracted from 2016 to 2019, and the ICD codes used in this study are described in Table 1.

**Table 1:** ICD codes used for the analysis. ICD: International Classification of Disease: SSI: Surgical site infection.

Comorbidities Codes	Medical Complication Codes	Surgical Complication Codes
Diabetes without complications: E119	Acute Renal Failure: N170, N171, N172, N178, N179	Periprosthetic fracture: T84010A, T84011A, T84012A, T84013A, T84018A, T84019A, M9665, M96661, M96662, M96669, M96671, M96672, M96679, M9669, M9701XA, M9702XA, M9711XA, M9712XA
Diabetes with complications: E1169	Myocardial Infarction: I2101, I2102, I2111, I2113, I12114, I12119, I2121, I12129, I21A1	Periprosthetic dislocation: T84020A, T84021A, T84022A, T84023A, T84028A, T84029A
Tobacco-related disorder: Z87891	Blood loss anemia: D62	Periprosthetic mechanical complications: T84090A, T84091A, T84092A, T84093A, T84098A, T84099A
Obesity: E660, E6601, E6609, E661, E662, E668, E669, Z6830, Z6831, Z6832, Z6833, Z6834, Z6835, Z6836, Z6837, Z6838, Z6839	Pneumonia: J189, J159, J22	Periprosthetic infection: T8450XA, T8451XA, T8452XA, T8453XA, T8454XA, T8459XA
Morbidly Obese: Z6841, Z6842, Z6843, Z6844, Z6845	Blood transfusion: 30233N1	Superficial SSI: T8141XA
	Pulmonary embolism: I2602, I2609, I2692, I2699	
	DVT: I82401, I82402, I82403, I82409, I82411, I82412, I82413, I82419, I82421, I82422, I82423, I82429, I82431, I82432, I82433, I82439, I82441, I82442, I82443, I82449, I82491, I82492, I82493, I82499, I824Y1, I824Y2, I824Y3, I824Y9, I824Z1, I824Z2, I824Z3, I824Z4	
	Cirrhosis: K7400, K7401, K7402, K7402, K741 K742, K743, K744, K745, K746, K7460, K7469	

Data acquisition

The study received an exemption from the IRB as it utilized de-identified data that is publicly available. This study included all patients with ICD-10 and Clinical Modification/Procedure Coding System (CMP) codes for TKA. Patients were split up into two groups: Cirrhosis and non-cirrhosis (control). Our demographic analysis considered age, sex, ethnicity, and the presence of obesity. Complications following surgery, such as postoperative anemia, hypotension, acute renal failure, deep vein thrombosis (DVT), and pulmonary embolism (PE), were also considered. In addition to systemic medical effects, including myocardial infarction (MI) and pneumonia, our investigation took into account regional problems such as periprosthetic infections, prosthetic dislocations, and periprosthetic fractures. Finally, details on each patient's overall LOS in the hospital and associated medical expenses were acquired.

Statistical analysis

All statistical analyses were performed using SPSS version 27.0 (IBM Corp., Armonk, NY, USA). Descriptive statistics were used to construct the patients' demographic data. Both matched and unmatched analyses were conducted. A 1:1 propensity matching algorithm was employed, considering preoperative factors such as age, sex, race, obesity, and diabetes (with and without complications). T-tests were used to examine the numerical variables. The Chi-square technique was used to evaluate binomial variables. Fisher's exact test was used when the incidence values were under 5. A p-value of less than 0.05 was deemed statistically significant for all tests. We calculated the odds ratios and their corresponding 95% CIs for surgical outcomes and complications by comparing the incidence rates in the cirrhosis group to those in the control group.

## Results

The NIS database identified 558,256 patients who underwent TKA between 2016 and 2019. Within this sample, only 1670 patients (0.3%) had cirrhosis, while the rest were control patients who did not have cirrhosis.

Patient demographics

Of the cirrhosis group, the average age was 65.91 ± 8.29 years, and 60.1% (N=1003) of the patients were female. Although there was no major gender disparity between the two groups, it was observed that those with cirrhosis were younger, and this difference was statistically significant (mean age: 65.9 vs. 66.7 years, P<0.001). Besides, the cirrhotic group demonstrated a higher prevalence of diabetes mellitus (18.9% vs. 14.8%, P<0.001) and obesity (37.8% vs. 30.9%, P<0.001). In comparison, a significantly higher rate of tobacco use was noted in the control population (16% vs. 12%, P<0.001) (Table 2).

**Table 2:** Patient demographics before propensity matching.

	Cirrhosis group (1670)	Control group (556586)	Significance
Mean Age (standard deviation) in years	65.91 (8.29%)	66.72 (8.29%)	P < 0.001
Sex (proportion female)	1003 (60.1%)	342417 (61.5%)	P = 0.220
Diabetes without complication (proportion diabetic)	315 (18.9%)	82149 (14.8%)	P < 0.001
Tobacco Use Disorder (proportion users)	207 (12.4%)	88162 (15.8%)	P < 0.001
Obesity (proportion obese)	628 (37.8%)	172033 (30.9%)	P < 0.001

Unmatched postoperative outcomes analysis

Unmatched analyses revealed that the cirrhosis group patients had a much higher likelihood of mortality than the control group (OR: 5.13, 95% CI: 1.64-16.08, p=0.02). Moreover, acute renal failure (OR: 3.33, 95% CI: 2.73-4.06, p<0.001), blood loss anemia (OR: 1.57, 95% CI: 1.39-1.76, p<0.001), pneumonia (OR: 3.42, 95% CI: 1.88-6.20, p<0.001), periprosthetic fracture (OR: 2.86, 95% CI: 1.84-4.46, p<0.001), periprosthetic infection (OR: 4.71, 95% CI: 3.75-5.92, p<0.001), and blood transfusion (OR: 3.24, 95% CI: 2.57-4.08, p<0.001), were all more likely to occur in the cirrhosis group in comparison to the control group (Table 3). The ORs of the rest, comprising wound dehiscence, mechanical complications of the implants, DVT, PE, and MI, did not demonstrate statistical significance.

**Table 3:** Unmatched analysis: Post-operative complications. *Exact number not reported if value between 1 and 10 as per Healthcare Cost and Utilization Project (HCUP) Data Use Agreement (DUA).

Post-operative Variables	Cirrhosis group (1670)	Control group (556586)	Odds Ratio (Cirrhosis group/Control group)	Odds Ratio 95% CI	Significance
Mortality	*0.2%	195 (<0.0003%)	5.13	(1.64, 16.08)	P = 0.02
Acute Renal Failure	105 (6.3%)	10980 (2%)	3.33	(2.73, 4.06)	P < 0.001
Myocardial Infarction	0	109 (0.0002%)	NA	NA	P = 0.567
Blood Loss Anemia	369 (22.1%)	85188 (15.3%)	1.57	(1.39, 1.76)	P < 0.001
Pneumonia	11 (0.7%)	1077 (0.2%)	3.42	(1.88, 6.20)	P < 0.001
Pulmonary Embolism	*0.1%	1236 (0.2%)	0.53	(0.13, 2.15)	P = 0.375
Deep Vein Thrombosis	*0.3%	1257 (0.2%)	1.32	(0.55, 3.19)	P= 0.527
Periprosthetic Fracture	20 (1.2%)	2343 (0.4%)	2.86	(1.84, 4.46)	P<0.001
Periprosthetic Mechanical Complication	20 (1.2%)	4484 (0.8%)	1.49	(0.96, 2.32)	P = 0.674
Periprosthetic infection	78 (4.7%)	5724 (1%)	4.71	(3.75, 5.92)	P <0.001
Wound Dehiscence	0.2%	527 (0.1%)	1.89	(0.61, 5.91)	P = 0.260
Blood Transfusion	77 (4.6%)	8177 (1.5%)	3.24	(2.57, 4.08)	P <0.001

The LOS analyses revealed that before matching, the cirrhosis group had a significantly longer stay than the control group (3.21 vs. 2.34 days, p<0.001). After matching, the difference remained statistically significant but with slight attenuation (4.22 vs 3.68 days, p=0.016). Likewise, the cirrhosis group incurred a higher COC before matching ($81,282) and after matching ($90,624) than the patients in the control group (p<0.001) (Table 4).

**Table 4:** Length of stay and cost of care in the unmatched and matched cohorts.

	Unmatched Cirrhosis Group	Unmatched Control Group	P-value	Matched Cirrhosis Group	Matched Control Group	P-value
Length of stay	3.21 (4.04)	2.34 (1.92)	<0.001	4.22 (5.86)	3.68 (4.56)	P=0.016
Hospital charges	81282 (96,932)	64761 (45,586)	<0.001	90624 (78180.84)	80676.87 (66353.87)	P<0.001

Matched postoperative outcomes analysis

Matched information regarding both groups can be found in Table 5. After matching, there was significant attenuation as patients in the cirrhosis group had a greater propensity for only acute renal failure (OR: 3.05, 95% CI: 2.07-4.50, p<0.001), blood loss anemia (OR: 1.60, 95% CI: 1.34-1.92, p<0.001), periprosthetic fracture (OR: 3.27, 95% CI: 1.31-8.18, p=0.007), periprosthetic infection (OR: 3.14, 95% CI: 1.99-4.95, p<0.001), and blood transfusions (OR: 1.62, 95% CI: 1.12-2.35, p=0.009). There were no significant differences between the groups regarding mortality, MI, pneumonia, PE, DVT, periprosthetic mechanical complication, and wound dehiscence (Table 6).

**Table 5:** Patient demographics of matched cohort *Exact number not reported if value between 1 and 10 as per Healthcare Cost and Utilization Project (HCUP) Data Use Agreement (DUA).

	Cirrhosis group (1627)	Control group (1628)	Significance
				Age (SD)	64.57 (10.0)	64.89 (11.04)	P = 0.472
Sex (proportion female) *	1003 (60.1%)	979 (60.1%)	p = 0.965
Diabetes without complications (proportion diabetic)	315 (18.9%)	312 (19.2%)	P = 0.825
Tobacco Use Disorder (proportion users) *	207 (12.4%)	200 (12.3%%)	P = 0.919
Obesity (proportion obese)	628 (37.8%)	615 (37.6%)	P = 0.919
Race			p = 0.563
White	1253 (77.0%)	1255 (76.9%)	
Black	125 (7.7%)	124 (7.6%)	
Hispanic	173 (10.6%)	173 (10.6%)	
Asian	24 (1.5%)	24 (1.5%)	
Native American	*0.60%	*0.60%	
Age Categorical			P= 1.000
<60	357 (21.4%)	346 (21.3%)	
60 to 70	774 (46.3%)	757 (46.5%)	
70 to 80	435 (26.7%)	447 (26.8%)	
80 to 90	89 (5.5%)	91 (5.4%)	
>90	* 0.10%	*0.10%	

**Table 6:** Matched sample analysis: post-operative complications. * Exact number not reported if value between 1 and 10 as per Healthcare Cost and Utilization Project (HCUP) Data Use Agreement (DUA).

Post Operative Variables	Cirrhosis group (1670)	Control group (1628)	Odds Ratio (Cirrhosis group/Control group)	Odds Ratio 95% CI	Significance
Mortality	*0.2%	*0.1%	1.46	0.24-8.76	0.675
Acute Renal Failure	105 (6.3%)	35 (2.1%)	3.05	2.07-4.50	<0.001
Myocardial Infarction	0	*0.1%	0.49	0.47-0.51	0.31
Blood Loss Anemia	369 (22.1%)	294 (15%)	1.60	1.34-1.92	<0.001
Pneumonia	11 (0.7%)	*0.2%	2.69	0.85-8.47	0.078
Pulmonary Embolism	*0.1%	*0.4%	0.278	0.05-1.33	0.08
Deep Vein Thrombosis	*0.3%	*0.4%	0.81	0.24-2.66	0.731
Periprosthetic Fracture	20 (1.2%)	*0.4%	3.27	1.31-8.18	0.007
Periprosthetic Mechanical Complication	20 (1.2%)	0.6%)	1.96	0.94-4.20	0.07
Periprosthetic Infection	78 (4.7%)	25 (1.5%)	3.14	1.99-4.95	<0.001
Wound Dehiscence	0.2%	0.4%	0.48	0.12-1.94	0.298
Blood Transfusion	77 (4.6%)	47 2.9%)	1.62	1.12-2.35	0.009

## Discussion

In the present research, an assessment of the in-hospital outcomes of 558,256 adult patients undergoing primary TKA revealed that liver cirrhosis was associated with higher rates of certain perioperative complications. Specifically, the odds of developing acute renal failure, periprosthetic fracture, and prosthetic joint infections were three times higher at a minimum. Further, the postoperative hospital stays of cirrhotic patients were approximately one day longer, and as a consequence, the overall costs were around $10,000 greater than those of non-cirrhotic patients. Despite the five-fold increase in mortality and three-fold increase in pneumonia among cirrhotic patients, no significant differences were observed after matching. As is well known, the liver plays a crucial role in developing and maturing various proteins, including those involved in the coagulation cascade and acute phase reactants [19]. As a consequence, patients with cirrhosis have increased rates of complications with numerous surgical procedures, including total joint arthroplasties [17,21]. A study by Shih LY et al. reported that cirrhotic TKA patients had increased blood loss, longer hospital stays, and higher mortality rates than control patients, with p-values less than 0.006 for each [12]. Bell JE et al. reviewed the Medicare database with a minimum one-year follow-up. They concluded that cirrhotic patients who have undergone TKA have about three times higher risk of disseminated intravascular coagulation (p= 0.003) and hepatic encephalopathy (p < 0.001), with prosthetic joint infection (p < 0.001) risk being about double than that of the control population [19]. In a study using the NIS database, Newman et al. reported that patients with cirrhosis had a higher likelihood of complications following TKA, with an odds ratio of 1.55 (95% CI: 1.47-1.63), and THA, with an odds ratio of 1.59 (95% CI: 1.50-1.69). Additionally, TKA patients with cirrhosis incurred additional costs of $1,857 and an increased length of stay (LOS) by 0.30 days. In comparison, THA patients with cirrhosis faced extra costs of $1,497 and an extension of LOS by 0.48 days [22].

Thus, it is imperative to recognize this select patient group's unique medical and surgical issues, especially how to optimize controllable factors [2,23]. The results of our study presented the highest odds, more than three-fold, for three distinct complications comprising acute renal failure, periprosthetic fracture, and prosthetic joint infection. Anemia caused by blood loss and the need for blood transfusion was 1.5 times more likely than in the control group. This data is indispensable to construct a multi-disciplinary strategy with adequate pre-, intra-, and postoperative considerations to maximize outcomes. Similarly, our previous study found that dialysis patients also had increased perioperative complications to consider [24]. A significant factor contributing to high medical expenses is the LOS, so total lower joint arthroplasty patients with comorbidities face higher costs. It has been estimated that cirrhotic TKA patients incurred a $1857 higher cost and 0.30 days extra LOS, while cirrhotic THA patients experienced $1497 more costs and 0.48 days extra LOS [22]. According to a recent NIS study, patients with cirrhosis who underwent primary hip arthroplasty spent an additional $10,000 compared to the control group, a statistically significant difference (p=.001) [21]. Likewise, Medicare patients with cirrhosis had longer hospital stays (3.57 vs. 3.23, p< 0.001) and higher 90-day charges ($76,082 vs. $56,940, 0.001) after having total knee replacement compared to those without cirrhosis, according to a recent study [19]. The results of the present study are in agreement, showing extra costs ($90,634 vs. $80,676) during their hospital stays after a TKA besides a longer LOS (4.22 vs. 3.68), both of which were statistically significant. The data we have obtained in the recent four years could be employed as a reasonable cost estimation for healthcare centers in today's bundled payment plans.

This study has noteworthy strengths and shortcomings that deserve consideration. Being an administrative database, the NIS is prone to sampling bias because of potential data entry errors, missing information, and coding discrepancies. Despite the abundant patient data, the lack of specific clinical details, lab investigators, and patient outcomes following discharge can affect the conclusions. Without factoring in outpatient treatment and postoperative readmissions, the NIS data is inadequate to determine late-term morbidities and fatalities. Besides, the available inpatient data does not offer insights into major intraoperative events and complications, which could impact the LOS and hospital costs. Finally, as a national estimate, the geographic granularity may not apply to regional or local research or certain demographic groups, limiting its generalizability.

Nevertheless, due to its extensive sample size, the NIS data closely represents the US population and serves as an invaluable resource for understanding national healthcare trends and disparities. Our study, spanning the years 2016 to 2019, presents the most comprehensive yet cost-effective approach for analyzing the effects of cirrhosis among TKA patients in the trending era of outpatient joint replacement procedures and bundled payment reimbursement systems.

## Conclusions

To the best of our knowledge, this is the first large-database study that has examined perioperative complications and hospital admission characteristics of cirrhotic patients following TKA. Cirrhosis is linked to extended hospital stays, increased medical expenses, and a higher likelihood of perioperative in-hospital complications following TKA, such as anemia due to blood loss, acute renal failure, and periprosthetic fractures and infection. This information can be valuable for preoperative patient counseling and enhancing healthcare resource allocation without compromising patient well-being and financial reimbursement from payers. When assessing hospital expenditures for cirrhotic patients, it is crucial to consider the higher rate of perioperative problems in this patient group. This data will help providers make informed decisions about patient care and resource utilization for cirrhotic patients undergoing TKA.

## References

[1] Kaltenbach MG, Mahmud N (2023). Assessing the risk of surgery in patients with cirrhosis. Hepatol Commun.

[2] Newman KL, Johnson KM, Cornia PB, Wu P, Itani K, Ioannou GN (2020). Perioperative evaluation and management of patients with cirrhosis: risk assessment, surgical outcomes, and future directions. Clin Gastroenterol Hepatol.

[3] Wiegand J, Berg T (2013). The etiology, diagnosis and prevention of liver cirrhosis: part 1 of a series on liver cirrhosis. Dtsch Arztebl Int.

[4] Schuppan D, Afdhal NH (2008). Liver cirrhosis. Lancet.

[5] GBD 2017 Cirrhosis Collaborators (2020). The global, regional, and national burden of cirrhosis by cause in 195 countries and territories, 1990-2017: a systematic analysis for the Global Burden of Disease Study 2017. Lancet Gastroenterol Hepatol.

[6] Orman ES, Roberts A, Ghabril M, Nephew L, Desai AP, Patidar K, Chalasani N (2019). Trends in characteristics, mortality, and other outcomes of patients with newly diagnosed cirrhosis. JAMA Netw Open.

[7] Asrani SK, Devarbhavi H, Eaton J, Kamath PS (2019). Burden of liver diseases in the world. J Hepatol.

[8] Golabi P, Paik JM, AlQahtani S, Younossi Y, Tuncer G, Younossi ZM (2021). Burden of non-alcoholic fatty liver disease in Asia, the Middle East and North Africa: data from Global Burden of Disease 2009-2019. J Hepatol.

[9] Liu YB, Chen MK (2022). Epidemiology of liver cirrhosis and associated complications: current knowledge and future directions. World J Gastroenterol.

[10] Huang DQ, Terrault NA, Tacke F, Gluud LL, Arrese M, Bugianesi E, Loomba R (2023). Global epidemiology of cirrhosis - aetiology, trends and predictions. Nat Rev Gastroenterol Hepatol.

[11] Child CG, Turcotte JG (1964). Surgery and portal hypertension. Major Probl Clin Surg.

[12] Shih LY, Cheng CY, Chang CH, Hsu KY, Hsu RW, Shih HN (2004). Total knee arthroplasty in patients with liver cirrhosis. J Bone Joint Surg Am.

[13] Sloan M, Premkumar A, Sheth NP (2018). Projected volume of primary total joint arthroplasty in the U.S., 2014 to 2030. J Bone Joint Surg Am.

[14] Schwartz AJ, Chang YH, Bozic KJ, Etzioni DA (2019). Evidence of pent-up demand for total hip and total knee arthroplasty at age 65. J Arthroplasty.

[15] Lu Y, Lin CC, Stepanyan H (2020). Impact of cirrhosis on morbidity and mortality after spinal fusion. Global Spine J.

[16] Tseng FJ, Gou GH, Wang SH, Shyu JF, Pan RY (2022). Chronic liver disease and cirrhosis increase morbidity in geriatric patients treated surgically for hip fractures: analysis of the US Nationwide Inpatient Sample. BMC Geriatr.

[17] Parikh ND, Chang YH, Tapper EB, Mathur AK (2019). Outcomes of patients with cirrhosis undergoing orthopedic procedures: an analysis of the nationwide inpatient sample. J Clin Gastroenterol.

[18] Rozell JC, Courtney PM, Dattilo JR, Wu CH, Lee GC (2017). Late complications following elective primary total hip and knee arthroplasty: who, when, and how?. J Arthroplasty.

[19] Bell JE, Amin R, Labaran LA, Sequeira SB, Rao SS, Werner BC (2021). Impact of compensated cirrhosis etiology on postoperative outcomes following total knee arthroplasty. J Arthroplasty.

[20] (2023). HCUP-US NIS Overview. http://us.ahrq.gov/nisoverview.jsp.

[21] Cheppalli NS, Metikala S, Beale J, Mounsamy V, Sambandam S (2023). Total hip arthroplasty in cirrhosis is associated with increased complications during the hospital stay, length of stay, and cost of care: a propensity matched database study. Arch Bone Jt Surg.

[22] Newman JM, Schiltz NK, Mudd CD, Szubski CR, Klika AK, Barsoum WK (2016). Impact of cirrhosis on resource use and inpatient complications in patients undergoing total knee and hip arthroplasty. J Arthroplasty.

[23] Onochie E, Kayani B, Dawson-Bowling S, Millington S, Achan P, Hanna S (2019). Total hip arthroplasty in patients with chronic liver disease: a systematic review. SICOT J.

[24] Venishetty N, Wukich DK, Beale J, Riley Martinez J, Toutoungy M, Mounasamy V, Sambandam S (2023). Total knee arthroplasty in dialysis patients: a national in-patient sample-based study of perioperative complications. Knee Surg Relat Res.

